# Effects of osteoblast-derived extracellular vesicles on aggressiveness, redox status and mitochondrial bioenergetics of MNNG/HOS osteosarcoma cells

**DOI:** 10.3389/fonc.2022.983254

**Published:** 2022-12-05

**Authors:** Marco Ponzetti, Argia Ucci, Chiara Puri, Luca Giacchi, Irene Flati, Daria Capece, Francesca Zazzeroni, Alfredo Cappariello, Nadia Rucci, Stefano Falone

**Affiliations:** ^1^ Department of Biotechnological and Applied Clinical Sciences, University of L’Aquila, L’Aquila, Italy; ^2^ Department of Life, Health and Environmental Sciences, University of L’Aquila, L’Aquila, Italy

**Keywords:** osteosarcoma, extracellular vesicles, redox status, bioenergetics, tumour microenvironment, apoptosis, mitochondria, cell communication

## Abstract

Osteosarcoma is the most common primary bone malignancy. The crosstalk between osteosarcoma and the surrounding tumour microenvironment (TME) drives key events that lead to metastasization, one of the main obstacles for definitive cure of most malignancies. Extracellular vesicles (EVs), lipid bilayer nanoparticles used by cells for intercellular communication, are emerging as critical biological mediators that permit the interplay between neoplasms and the tumour microenvironment, modulating re-wiring of energy metabolism and redox homeostatic processes. We previously showed that EVs derived from the human osteosarcoma cells influence bone cells, including osteoblasts. We here investigated whether the opposite could also be true, studying how osteoblast-derived EVs (OB-EVs) could alter tumour phenotype, mitochondrial energy metabolism, redox status and oxidative damage in MNNG/HOS osteosarcoma cells.These were treated with EVs obtained from mouse primary osteoblasts, and the following endpoints were investigated: i) cell viability and proliferation; ii) apoptosis; iii) migration and invasive capacity; iv) stemness features; v) mitochondrial function and energy metabolism; vi) redox status, antioxidant capacity and oxidative molecular damage. OB-EVs decreased MNNG/HOS metabolic activity and viability, which however was not accompanied by impaired proliferation nor by increased apoptosis, with respect to control. In addition, OB-EV-treated cells exhibited a significant reduction of motility and *in vitro* invasion as compared to untreated cells. Although the antioxidant N-acetyl-L-cysteine reverted the cytotoxic effect of OB-EVs, no evidence of oxidative stress was observed in treated cells. However, the redox balance of glutathione was significantly shifted towards a pro-oxidant state, even though the major antioxidant enzymatic protection did not respond to the pro-oxidant challenge. We did not find strong evidence of mitochondrial involvement or major energy metabolic switches induced by OB-EVs, but a trend of reduction in seahorse assay basal respiration was observed, suggesting that OB-EVs could represent a mild metabolic challenge for osteosarcoma cells. In summary, our findings suggest that OB-EVs could serve as important means through which TME and osteosarcoma core cross-communicate. For the first time, we proved that OB-EVs reduced osteosarcoma cells’ aggressiveness and viability through redox-dependent signalling pathways, even though mitochondrial dynamics and energy metabolism did not appear as processes critically needed to respond to OB-EVs.

## Introduction

Osteosarcoma is the most common primary bone tumour, especially in childhood and adolescence, representing 2% of all neoplasias in children up to 14 years of age, and 3% in those aged 14 to 19 (1, 2). The consequences of osteosarcoma are devastating, ranging from limb amputation (20% of operable osteosarcomas) to death because of lung metastases (3), not to mention the physical and psychological consequences on both children and their families. Osteosarcoma derives from a malignant transformation of mesenchymal cells and is characterised by deposition of an osteoid-like matrix, with varying degrees of mineralization that can be studied by x-ray (4). Drug resistance is a serious issue in osteosarcoma, owing to its high genetic plasticity, which eventually leads to lung metastasization, inevitably ending with the death of the patient (5–7). Treatment of osteosarcoma often relies on reactive oxygen species (ROS)-generating compounds, and an important role in osteosarcoma chemoresistance has been recently acknowledged for improved protection against redox imbalance and ROS over-production (8, 9). Cancer cells are known to show a strong antioxidant machinery that serves to counteract high ROS basal levels, which are linked to mitochondrial metabolic reprogramming and to ROS-mediated signalling promoting proliferation (10). In this context, some authors have reported a critical role for mitochondrial dysfunction in the cytotoxic effect elicited by redox-active compounds that are potentially relevant for clinical treatment of osteosarcoma (11), and others have demonstrated that chemoresistant osteosarcoma cells exhibit lower mitochondrial activity with respect to non resistant cells (12). Today, it is widely accepted that cancer cell phenotype is influenced by the tumour microenvironment (TME), which represents a complex ecosystem that includes tumour cells, immune cells and stromal cells. In particular, the interactions among the components of the TME control the tumour’s fate, along with its aggressiveness and resistance against therapies (13). Extracellular vesicles (EVs), that consist of lipid bilayer nano/microparticles secreted by all cell types, are emerging as a powerful means of communication between osteosarcoma and the TME. EVs contain a plethora of bioactive macromolecules, including miRNAs, membrane and luminal proteins, lncRNAs, circRNAs, tRNAs, many of which exhibit target specificity, being uptaken by specific cell types mainly depending on their membrane proteins (14, 15). The importance of EVs in cancer is starting to be well recognised, thanks to pioneering works from the late 2010s (16–19), yet their importance in primary bone cancers has not been elucidated in detail. Some of us previously demonstrated that EVs derived from the human osteosarcoma cell line MNNG/HOS were able to influence bone cells (20), especially osteoblasts. However, whether osteoblasts are also able to influence osteosarcoma phenotype through EVs still needs to be fully elucidated. In this work, we aimed at contributing to this field, focusing our attention on the effects induced by OB-EVs in MNNG/HOS cells, in terms of tumour phenotype, energy metabolism, redox status and oxidative damage.

## Materials and methods

### Materials

Dulbecco’s modified Minimum Essential Medium (DMEM), Dulbecco’s PBS (DPBS), Foetal Bovine Serum (FBS), penicillin, streptomycin and trypsin were supplied by GIBCO (Uxbridge, UK). All sterile plasticware was from Falcon Becton-Dickinson (Cowley, Oxford, UK) or Costar (Cambridge, MA, USA). The Matrigel Matrix (cat. no. 354262) was purchased from Corning (NY, USA), the EdU (5- ethynyl-2’-deoxyuridine)-HTS Kit 488 (cat. no. BCK-HTS488) was provided by Base Click GmBH (Munich, Germany). Trypan blue 0.4% (cat. no. 15250061), Mitotracker green FM (cat. no. M7514) and RevertAid First Strand cDNA Synthesis (cat. no. K1622) were from Thermo Scientific (Waltham, MA, USA). The glutathione assay kit (cat. no. 703002) and the TBARS Assay Kit (cat. no. 10009055) were supplied by the Cayman Chemical Company (Ann Arbor, MI, USA). The human MMPs array C1 kit (cat. no. AAH-MMP-1-8) was supplied from RayBiotech (Peachtree Corners, GE, USA), Bradford assay (cat. no. A6932,0500) was from Panreac Applichem (Darmstadt, Germany). All other reagents, including 3-(4,5-dimethylthiazol-2-yl)-2,5-diphenyltetrazolium (MTT) bromide reduction assay, staurosporine (cat. no. 569396), N-acetyl-L-cysteine (L-NAC; cat. A7250), etomoxir (cat. E1905), oligomycin A (cat. 75351), 2,4-dinitrophenol (2,4-DNP; cat. D198501), rotenone (cat. R8875), antimycin A (cat. A8674), NADPH (cat. N7505) and GSSG (cat. G4376) were supplied by Merck Life Science (Milan, Italy). Dithiothreitol (DTT; cat. D1532) was supplied by Invitrogen (Waltham, MA, USA). The L.D.H. (LDH-P) DGKC method-based kit (cat. K2011) was supplied by Biolabo (Maizy, France). Antibodies used in this work are summarised in **Supplementary Table 1**.

### Animal ethical approval

All procedures involving animals and their care was conducted in conformity with national and international laws and policies (European Economic Community Council Directive 86/609, OJ L 358, 1, December 12, 1987; Italian Legislative Decree no. 26, Gazzetta Ufficiale della Repubblica Italiana no. 61, March 4th, 2014; guide for the Care and Use of Laboratory Animals, National Institute of Health, Publication no. 85-23, 1985) and the Animal Research: Reporting of *in Vivo* Experiments (ARRIVE) guidelines. Animal procedures received Institutional approval by the Italian Ministry of Health (approval no. 622/2021-PR).

### Cell cultures

The human osteosarcoma cell line MNNG/HOS (RRID : CVCL_0439) was obtained from the European Collection of Authenticated Cell Cultures (ECACC, Salisbury, UK) and grown at 37 °C, 5 % CO2 in DMEM supplemented with 10% FBS, 100 IU/ml penicillin, 100 μg/ml streptomycin, and 2 mM L-glutamine.

### Osteoblast primary cultures

Calvariae from 7-day-old CD1 mice were explanted, cleaned free of soft tissues and digested three times with 1 mg/ml *Clostridium histolyticum* type IV collagenase and 0.25% trypsin, for 15, 30 and 45 min, respectively, at 37°C with gentle agitation. Cells from the second and third digestions were plated following centrifugation at 300 x *g* for 7 min and grown at 37°C, 5% CO2 in DMEM plus 10% FBS. At confluence, cells were trypsinised and plated according to the experimental protocol. The purity of the culture was evaluated by the transcriptional expression of the osteoblast biomarkers Alkaline Phosphatase (ALP), Runt-related transcription factor 2 (*Runx2*), type I collagen and osteocalcin and by the histochemical evaluation of ALP activity.

### Extracellular vesicles isolation

Osteoblast derived EVs (OB-EVs) were isolated according to Ucci et al. (20) and Loftus et al. (21). Briefly, upon reaching 80% confluence, mouse primary osteoblasts were washed in DPBS and starved in serum-free DMEM to prevent contamination from FBS-EVs. After 24 h, the conditioned medium (CM) was collected and sequentially centrifuged at 300 x *g*, 4°C for 5 min to remove dead cells and at 5,000 x *g*, 4°C for 25 min to remove membrane debris. The supernatant was collected and transferred to a Beckman L7-65 ultracentrifuge in a Beckman SW41-Ti or SW28 rotor and centrifuged at 100,000 x *g*, at 9°C for 70 min. Supernatant was discarded, while the pellet, containing EVs, was resuspended in DMEM for cell treatments. To quantify OB-EVs, they were subjected to nanoparticle tracking analysis (see **Supplementary Figure 1A**) as well as to protein extraction, the latter giving a yield of 4.9 ± 1.3 µg/12 ml CM. Freshly-isolated EVs were used for all the subsequent experiments.

### Nanoparticle tracking analysis

EVs were isolated from osteoblast CM (12 ml collected from one 175cm^2^ flask, cell density = 3.5x10^4^ cells/cm^2^) and resuspended in 100µl of nanofiltered DPBS. EVs were then diluted 1:100 and used for nanoparticle tracking analysis using a nanosight NS300 NTA apparatus. Flow and camera gain were adjusted following the manufacturer’s instructions based on the NS300 quality control parameters. Five camera acquisitions of 60 seconds each were analysed for every biological replicate.

### Transmission electron microscopy

EVs were isolated from osteoblast CM (12 ml collected from one 175cm^2^ flask, cell density = 3.5x10^4^ cells/cm^2^) and resuspended in 2% PFA. Five µl of EVs were then put onto Formvar-coated grids and allowed to adsorb for 20 min in a dry environment. Grids were washed in PBS and fixed in 1% glutaraldehyde for 5 min. Samples were washed in distilled water and contrasted with 4% uranyl-oxalate solution for 5 min. Grids were air-dried for 10 min and observed under a Philips CM 30 TEM, 80 kV.

### EV internalisation assay

Extracellular vesicles derived from osteoblast CM (12 ml collected from one 175 cm^2^ flask, cell density= 3.5x10^4^ cells/cm^2^) were incubated at 37°C with the membrane-permeant green fluorescent dye 5-chloromethylfluoresceindiacetate (CMFDA) for 30 min followed by 5 min at 37°C with the red fluorescent membrane-labelling dye PKH26 (Sigma–Aldrich; #MINI26-1KT). Then, the EVs were washed in PBS and ultracentrifuged at 100,000 x *g*, at 4°C for 70 min. Finally, EVs were resuspended in PBS for the treatment. Target cells were incubated with stained or unstained EVs for 48h before microscopic assessment of internalisation. Nuclei were counterstained with DAPI.

### MTT assay

Metabolic activity of MNNG/HOS cells was assessed by the 3-(4,5-dimethylthiazol-2-yl)-2,5-diphenyltetrazolium (MTT) bromide reduction assay (22). Briefly, cells (9,000/cm^2^) were plated in 96-well plates, starved O/N in serum-free DMEM and then treated with OB-EVs (isolated from 12 ml of CM collected from one 175-cm^2^ flask to treat 9 wells). MTT was dissolved in DPBS at 5 mg/ml concentration, and added at 1:6 (v/v) ratio directly into the cell supernatant. Three hours later, medium was removed and DMSO was added to dissolve the precipitated formazan salts arising from the reaction. Plates were shaken at 160 rpm on an orbital shaker for 10 min, then absorbance at 595 nm was recorded and plotted as X-fold to the absorbance at t_0_.

### Viable cells counting

Viable cells counting was carried out through the Trypan blue exclusion test (23). MNNG/HOS cells were plated in 175-cm^2^ flasks and treated with DMEM (control), OB-EVs (isolated from 36 ml of CM collected from three 175-cm^2^ flask, target cell surface:isolation surface ratio=1:3) or OB-EVs + 5 mM L-NAC. Twenty-four hours later, cells were detached using trypsin-EDTA and centrifuged at 400 x *g*. Pellets were resuspended in DPBS and a 20 µl aliquot was mixed with the same volume of 0.4% Trypan blue. Cells were incubated for 2 min at RT, then 10 µl of the cell suspension were transferred to an hemocytometer for viable cell counting. Cell viability was then expressed as % live cells/total cells.

### EdU (5- ethynyl-2’-deoxyuridine) cell proliferation assay

EdU cell proliferation assay was performed on MNNG/HOS cells pretreated with OB-EVs, by using the EdU (24) HTS Kit 488. Briefly, cells were plated onto cell culture dishes (90 mm Ø) and, upon reaching 80% of confluence, were starved O/N in serum-free DMEM and treated with OB-EVs isolated from 12 ml of CM collected from one 175-cm^2^ flask (target cell surface:isolation surface ratio=1:3) or with DMEM, as control. After 24 h, cells were detached and seeded in 96-well plates at a density of 9,000 cells/cm^2^), grown in standard conditions for 43 h and incubated for 5 hours with 5 μM EdU (48h total culture time). Cells were then washed in DPBS and fixed in 4% paraformaldehyde (PFA) for 10 min. EdU incorporation was detected using the EdU HTS Kit: cells were permeabilised with 0.5% Triton X-100 in PBS, incubated with the click assay cocktail for 30 min at RT, protected from light, and then washed twice in 1X rinse solution. Nuclei were counterstained with DAPI. Cell proliferation was assessed under a fluorescence microscope and expressed as the percentage of EdU-positive cells.

### Wound healing assay

MNNG/HOS cells were cultured in cell culture dishes (90 mm Ø) until confluence reached 80%. Then, cells were starved O/N and pre-treated for 24 hours with OB-EVs (previously isolated from 12 ml of CM collected from one 175-cm^2^ flask, target cell surface:isolation surface ratio=1:3) or with DMEM (control). Then, MNNG/HOS cells were detached and plated on 24 well-plates in DMEM+10% FBS. When confluence reached 100%, cell monolayers were scratched with a sterile tip to create a cross-shape wound (25). Pictures were taken at time 0 and 6 hours later under a phase-contrast microscope. Cell motility was evaluated by calculating the percentage of wound-healed area using NIH ImageJ software (RRID : SCR_003070).

### *In vitro* invasion assay

MNNG/HOS cells were plated in cell culture dishes (90 mm Ø) and, upon reaching 80% of confluence, they were washed twice in DPBS, starved overnight in serum-free DMEM and treated with OB-EVs (previously isolated from 12 ml of CM collected from one 175-cm^2^ flask, target cell surface:isolation surface ratio=1:3) or with DMEM, as control. After 24 h, MNNG/HOS cells were washed in DPBS detached by trypsin-EDTA, incubated for 30 min at 37°C in 5% CO_2_, and centrifuged at 300 x *g* for 5 min at RT. Each pellet was then resuspended in DPBS, centrifuged at 300 g for 5 min at RT and resuspended in serum-free DMEM. Then, 8 x 10^5^ cells were seeded in the upper compartment of each transwell, onto an 8.0 μm membrane pre-coated with Matrigel (26), while 0.8 ml of FBS were added to the lower compartment as chemoattractant. After 8 h, the cells that remained in the upper compartment were carefully removed using cotton swabs, while those migrated towards the lower compartment and remained in the matrigel were fixed with cold methanol, washed in PBS and stained with hematoxylin/eosin. Number of invaded cells per field was evaluated in seven fields/transwell.

### Oncosphere formation assays

MNNG/HOS cells were plated at a density of 4,000 cells/ml in oncosphere medium on poly-hydroxyethyl-methacrylate-coated plates (27). Oncosphere medium composition was as follows: DMEM/F12 with 1% penicillin/streptomycin, 20 nM progesterone, 100 μM putrescine, 1% insulin- transferrin-selenium A, 10 ng/ml basic fibroblast growth factor, 10 ng/ml epithelial growth factor with OB-EVs or DMEM as control (27). Growth factors and OB-EVs (EVs isolated from 12 ml of CM collected from one 175-cm^2^ flask and then added at 3.85cm^2^ isolation surface/ml of medium ratio) of EVs were refreshed twice a week. After 7 and 10 days, pictures of the whole plates were taken with an inverted phase contrast microscope equipped with a CMOS sensor camera, and images were analysed by imageJ to evaluate oncosphere number and area.

### Western blotting

MNNG/HOS cells were plated in cell culture dishes (90 mm Ø) and, upon reaching 80% of confluence, they were washed twice in DPBS, starved overnight in serum-free DMEM and treated with OB-EVs (previously isolated from 12 ml of CM collected from one 175-cm^2^ flask, target cell surface:isolation surface ratio=1:3) or with DMEM, as control. After 24 h of treatment, cells were lysed in RIPA buffer (50 mM Tris HCl pH 7.5, 150 mM NaCl, 1% Nonidet P-40, 0.5% sodium deoxycholate, 0.1% SDS) containing protease inhibitors. Mouse primary osteoblasts and EVs were also subjected to protein extraction as described above. Proteins (8 µg for osteoblasts and OB-EVs and 30µg for MNNG/HOS cells) were resolved by 10-12% SDS-PAGE and transferred to nitrocellulose membranes. Blots were incubated 1 hour in 5% nonfat dry milk in TBS-T, probed with the primary antibody (**Supplementary Table 1**) in 1% milk O/N at 4°C, washed and incubated with the appropriate HorseRadish Peroxidase (HRP)-conjugated secondary antibody (**Supplementary Table 1**) for 1 h at RT. After washing, protein bands were revealed by Enhanced ChemiLuminescence (ECL) and acquired using a Chemidoc XRS+ imaging system. The analysis of band intensities was performed using the NIH ImageJ tool (RRID : SCR_003070), and β-Actin or ɑ-Tubulin-normalised data were shown as fold *vs* DMEM (control).

### Matrix metalloproteinases protein array

MNNG/HOS cells were plated in cell culture dishes (90 mm Ø) and, upon reaching 80% of confluence, they were washed twice in DPBS, starved O/N in serum-free DMEM and treated with OB-EVs (previously isolated from 12 ml of CM collected from one 175-cm^2^ flask, target cell surface:isolation surface ratio=1:3) or with DMEM, as control. After 48 h, CM was collected, centrifuged at 300 x *g*, 4°C for 5 min, and subjected to the Human MMPs array C1 kit (RayBiotech, cat. no. AAH-MMP-1-8), following the manufacturer’s instructions. One ml of CM from MNNG/HOS untreated or treated with OB-EVs was incubated with each membrane, which includes 10 capture antibodies printed in duplicate. Membranes were washed and incubated with the Chemiluminescence Detection Buffer mix for the detection of the positive spots. For data analysis, the intensity of each spot was determined by densitometry using NIH ImageJ, and the average background subtracted. The intensity of positive control spots were used to normalise the signal intensity of the protein of interest. Data was shown as fold *vs* control (DMEM).

### Incucyte-based caspases 3/7 assay

MNNG/HOS cells were plated at a density of 9,000 cells/cm^2^ in 96-well plates and starved O/N. Then, cells were stained with a caspase 3/7-sensitive probe (Sartorius, cat. no. 4440) that crosses cell membranes but is only fluorescent in cells with active caspases 3/7, along with OB-EVs (isolated from 12 ml of CM collected from one 175-cm^2^ flask and then added to target cells at a target cells surface:isolation surface ratio=1:3), DMEM (control) or 5 µM staurosporine (positive control). After 30 min of incubation at RT, cells were transferred into a Sartorius Incucyte S3 live cells imager and captured (10X magnification) with a capture time interval of 2 h, across 48 h overall, using both phase contrast and green fluorescence channels. Data were analysed by Incucyte’s in-bundle software (rel. 2019B), and the metric used was the number of green positive objects (casp3/7^+^ cells)/confluence area. DMEM-normalised results were given including datasets from 4 independent experiments, with at least 6 technical replicates each.

### Incucyte-based Mitotracker assay

MNNG/HOS were plated at a density of 9,000 cells/cm^2^ in 96 well plates and starved O/N. Cells were then stained with MitoTracker green FM (Thermo Fisher, cat#M7514) following the manufacturer’s instructions and incubated at 37°C, 5% CO_2_ for 30 min. The staining solution was removed and cells were treated with OB-EVs (isolated from 12 ml of CM collected from one 175-cm^2^ flask and then added to target cells at a target cells surface:isolation surface ratio=1:3) or DMEM (control). Then, cells were transferred into a Sartorius Incucyte S3 live cells imager and images were captured (10X magnification) with a 2-hour time interval, across 48 hours overall, using both phase contrast and green fluorescence channels. Results were analysed using Incucyte’s in-bundle software (rel. 2019B), and the metric reported was the t_0_-normalised green fluorescent area/total cell area ratio. Datasets derived from 5 independent experiments, with at least 6 technical replicates.

### Glutathione assay

Cells were treated as described in the Viable cells counting section, then total (tGSH) and oxidised glutathione (GSSG) levels were measured using the method described by Baker and co-workers (28). GSSG was measured by first derivatizing GSH with 2-vinylpyridine (cat. no. 132292, Sigma–Aldrich), as recommended by the manufacturer. Briefly, DMEM-, OB-EV- and OB-EV+NAC-treated cells were homogenised in the MES buffer provided by the manufacturer (1.5 x 10^7^ cells/ml) and centrifuged at 10,000 x *g* for 15 min, at 4°C. Supernatants were immediately deproteinized with 5% (w/v) metaphosphoric acid (cat. no. 239275, Sigma–Aldrich), and centrifuged at 4,000 x *g* for 5 min. Protein-free supernatants (50 µl) and Assay Cocktail (150 µl) were mixed in a 96-well microplate, and the colour development was followed in a Victor3 microplate reader (PerkinElmer Inc., Waltham, MA, USA) at 405 nm for 30 min with 5-min time intervals. Calibration curves were obtained from pure GSSG- and GSH-containing reactions (range: 0-8 mM GSSG, 0-16 mM tGSH). All samples were blinded-processed in technical triplicates. Results were from 8 independent experiments.

### Thiobarbituric acid-reactive substances assay

Cells were treated as described in the Viable cells counting section, and then subjected to TBARS measurement, a well-established method used to detect lipid peroxidative damage (29, 30). Briefly, DMEM-, OB-EV- and OB-EV+NAC-treated cells were extracted in PBS (6 x 10^7^ cells/ml), by using three thaw/freeze cycles in liquid nitrogen. Samples were centrifuged at 16,000 x *g* for 30 min at 4°C. Supernatants (37.5 µl) were mixed with sodium dodecyl sulphate (SDS) (37.5 µl) and 1.5 ml of Colour Reagent, in triplicate, as suggested by the manufacturer. Then, reaction mixtures were incubated for 1 hour in boiling water and centrifuged at 1,600 x *g* for 10 min at 4°C. Supernatants were kept at RT for 5 min until clarified, and read at 532 nm in a Lambda 25 spectrophotometer (PerkinElmer Inc., Waltham, MA, USA). A linear calibration curve was obtained from pure malondialdehyde (MDA)-containing reactions (range: 0-50 mM). All samples were blinded-processed in technical replicates. Results were from 5 independent experiments.

### Catalase enzymatic activity assay

Cells were treated as described in the Viable cells counting section, harvested and lysed (2 x 10^7^ cells/ml) in a 100 mM phosphate buffer (pH 7), containing 0.1% (v/v) Triton X-100. Cell suspensions were lysed through N_2_-freezing and thawing (three cycles). Then, pellets were homogenised using 1.5 ml tube pestles for 2 min in ice. Samples were centrifuged at 16,000 x *g* for 30 min at 4 °C and the resulting supernatants were used both for Bradford assay to evaluate total protein concentration, using BSA as the standard, and for the assessment of the enzymatic activity of CAT (EC 1.11.1.6). The enzymatic activity of CAT was assayed by recording the disappearance of 10 mM hydrogen peroxide at 240 nm and 25 °C, as described by Aebi (31), using a Lambda25 spectrophotometer (PerkinElmer Inc., Waltham, MA, USA). One unit was defined as 1 µmol of hydrogen peroxide consumed/min. Three independent experiments were carried out with at least three technical replicates.

### Glutathione reductase enzymatic activity assay

Cells were treated as described in the Viable cells counting section, harvested and lysed (3 x 10^7^ cells/ml) in a 100 mM phosphate buffer (pH 7) supplemented with 2 mM EDTA and 3 mM DTT. Cell suspensions were lysed through N_2_-freezing and thawing (three cycles). Then, pellets were homogenised using 1.5 ml tube pestles for 2 min in ice. Samples were centrifuged at 16,000 x *g* for 30 min at 4 °C. The resulting supernatants were used for Bradford assay to evaluate total protein concentration, using BSA as the standard, and for the assessment of the enzymatic activity of GR (EC 1.6.4.27). The enzymatic activity of GR was assayed according to Di Ilio and colleagues (32), starting the reaction by adding GSSG (1.14 mM final concentration) and following the disappearance of 190 μM NADPH at 340 nm and 25 °C with a Lambda25 spectrophotometer (PerkinElmer Inc.). One unit was defined as 1 µmol of NADPH consumed/min. Five independent experiments were carried out with at least three technical replicates.

### Lactate dehydrogenase enzymatic activity assay

Cells were treated as described in the Viable cells counting section. Twenty-four hours later, conditioned media were collected and used for the assessment of the LDH enzymatic activity. The quantitative determination of LDH activity was assayed in duplicate using the L.D.H. (LDH-P) DGKC method-based kit by following the LDH-dependent decrease of NADH absorbance at 340 nm associated with reduction of pyruvate, as recommended by the manufacturer. Results from two independent experiments were analysed using a Kenza One analyzer (Biolabo), equipped with Kenza One v2.04 software.

### Seahorse-based assays

For bioenergetic profiling, 5,000 MNNG/HOS cells were seeded onto wells of Seahorse 96-well plates coated with 0.1% Collagen Type I. After 24 h cells were starved for further 24h and treated or not with OB-EVs (isolated from 12 ml of CM collected from one 175-cm^2^ flask and then added to target cells at a target cells surface:isolation surface ratio=1:3). After 24h cells were analysed by XF-96 Extracellular Flux Analyzer (Agilent Seahorse). ATP production rate was measured in DMEM XF Assay Medium (#103680-100, Agilent Seahorse) containing 1 mM pyruvate, 2 mM glutamine, and 10 mM glucose, following injection of 1.5 μM oligomycin A, 0.5 μM rotenone, and 0.5 μM antimycin A. Mitochondrial oxygen consumption rates (OCR) and glycolytic Extra-Cellular Acidification Rates (ECAR) were measured and then transformed into mitochondrial (mito-ATP) and glycolytic (glyco-ATP) ATP production rates, using validated algorithms provided in the Seahorse Agilent software. Mitostress test was conducted, and OCR was measured in the same media as above following injection of 1.5 μM oligomycin A, 75 μM 2,4-Dinitrophenol, 0.5 μM rotenone, and 0.5 μM antimycin A. Spare respiratory capacity (SRC) was calculated in accordance to the manufacturer’s specifications as the difference between maximal respiration (i.e., the maximum rate measurement recorded after 2,4-DNP injection after subtracting the non-mitochondrial respiration rate) and basal respiration (i.e., the last measurement recorded prior to oligomycin A injection after subtracting the non-mitochondrial respiration rate). Seahorse values were normalised by cell number well by well.

### Real time RT-PCR

Total RNA from osteoblast-derived EVs was extracted using TRIzol reagent, then RNA (1.5 μg) was reverse transcribed *via* a M-MLV reverse transcriptase-based first strand cDNA synthesis, as recommended by the suppliers. cDNA was subjected to real time PCR, using the Fast Advanced Master Mix (ThermoFisher Scientific, cat. 4444557), together with TaqMan^®^ Gene Expression Assays with IDs Mm00439154_m1 (*Mus musculus* glutathione reductase) and Mm00802658_m1 (*Mus musculus* glutamate-cysteine ligase catalytic subunit). Reactions (in triplicates) were set up in Primo^®^ FrameStar^®^ 96-well PCR plates (Euroclone, cat. ECPCR0770C), which were sealed with MicroAmp™ optical adhesive films (Applied Biosystems, cat. 4360954). The thermal profile of the Applied Biosystems VIIA7 was set as recommended by the manufacturer for absence/presence assays: 2 min at 50°C, 20 sec at 95°C, then 40 cycles with 1 sec at 95°C and 20 sec at 60°C, along with a final post-read stage of 30 sec at 60°C.

### Statistics

Results were expressed as means ± SEM. To compare curves in longitudinal studies, Graphpad Prism (RRID : SCR_002798, version 7.0) was used to run curve fitting tests and evaluate whether one curve could fit both the datasets compared. Shapiro-Wilk normality tests were performed to assess whether to use parametric or non-parametric tests. In experiments with more than 2 independent experimental groups, one-way ANOVA (parametric) or Kruskal-Wallis (non-parametric) was used to calculate statistical significance of differences. Unpaired Student’s t-test (parametric) or Mann-Whitney (non-parametric) tests were used when comparing 2 groups only. Dunn’s test was used when comparing non-normally distributed multiple groups with experimental pairing. Statistics used were specified in each figure legend. Differences were considered statistically significant when p was < 0.05.

## Results

### Characterisation of osteoblast-derived EVs

We first characterised the EVs isolated from mouse primary osteoblast conditioned media. EV size was confirmed by NanoSight, which also allowed to determine the EV concentration per preparation (**Supplementary Figure 1A**). The typical positive EV biomarkers, CD81, CD63 and Annexin II (*Anxa2*), were enriched in the EV protein lysates versus the source cells (**Supplementary Figure 1B**), while the mitochondrial protein SOD2, a possible negative marker for EVs, was barely detectable in EVs and well expressed in the cell protein lysate (**Supplementary Figure 1B**). The vesicular nature of the isolated particles was confirmed by TEM, which also showed the EV membrane integrity (**Supplementary Figure 1C**). Altogether, these results fulfil the requirements described in the MISEV2018 guidelines (33) to define a vesicular fraction as EVs.

**Figure 1:**
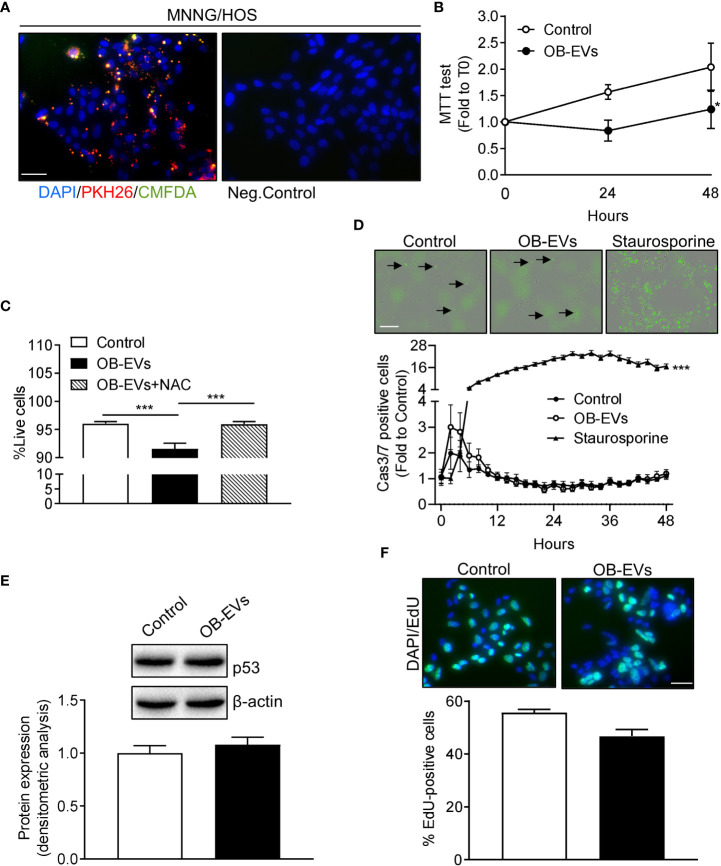
Effects of OB-EVs viability, apoptosis and proliferation of MNNG/HOS cells. **(A)** Left panel: representative fluorescence micrographs of MNNG/HOS cells incubated with osteoblast-derived EVs (OB-EVs) previously labelled with CMFDA (green cytoplasmic dye) and PKH26 (red lipophilic dye) for 48 hours. Right panel: representative image of the negative control performed on MNNG/HOS cells incubated with unlabeled OB-EVs. Cells were also stained with the nuclear dye DAPI. Data are representative of 3 independent preparations (scale bar=50 μm). **(B–E)** MNNG/HOS cells were plated and starved in serum-free DMEM O/N the next day. Then, cells were treated with DMEM (control), OB-EVs or OB-EVs + 5 mM N-acetyl-L-cysteine (NAC), for the time duration specified in the figures, or for 24 h when not specified. Cells were subjected to **(B)** MTT assay to assess cell viability/metabolic activity; **(C)** Trypan blue dye-exclusion test to evaluate the percentage of live cells; **(D)** Caspase 3/7 (Casp3/7) Incucyte-based assay for apoptosis (bar=200 µm, black arrows: apoptotic cells); **(E)** Western blotting to assess p53 protein expression/β-actin; **(F)** EdU-based assay to evaluate proliferation (scale bar=50 μm). **(B, E, F)** N=3, **(C)** N=8, **(D)** N=4. **(B, D)** Curve fitting test; **(C)** One-way ANOVA; **(E, F)** Unpaired Student’s *t*-test. *p<0.05; ***p<0.001. Data are presented as mean ± SEM.

We next investigated whether OB-EVs were actively internalised by MNNG/HOS cells. To this aim, OB-EVs were double-labelled with the intra-vesicular green-fluorescent dye, CMFDA, and with the red-fluorescent membrane dye, PKH26. MNNG/HOS cells were then incubated with these EVs for 48 hours. Fluorescence microscopy demonstrated that PKH26 and CMFDA fluorescence was detectable in osteosarcoma cells, suggesting internalisation of OB-EVs by the recipient cells (**Figure 1A**, left panel). Negative controls treated with unstained EVs confirmed specificity of the signal (**Figure 1A**, right panel).

### OB-EVs reduced MNNG/HOS cell viability without activating apoptosis or affecting cell proliferation

To assess whether OB-EVs could affect basic cellular activities, the first endpoints analysed were cell viability, death and proliferation. Interestingly, the MTT assay, which measures cell metabolic function, revealed a diminished activity of mitochondrial dehydrogenases in OB-EVs-treated MNNG/HOS cells, after 24 and 48 hours of treatment, as compared to control (**Figure 1B**). In order to investigate whether the reduction in MTT was accompanied by a reduced cell viability, we also performed a Trypan blue exclusion test, and found that OB-EVs reduced the percentage of live MNNG/HOS cells (**Figure 1C**). This result was confirmed by the increased extracellular levels of lactate dehydrogenase, a marker used to assess cell death (34), found in the medium of osteosarcoma cells treated with OB-EVs (+11.2% vs DMEM). Interestingly, the co-administration of the antioxidant N-acetyl-7L-cysteine (NAC) was able to revert the cytotoxic effect observed in OB-EV-treated cells (**Figure 1C**). To discriminate between necrotic and apoptotic death, we also performed an Incucyte-based time-course assay for detection of caspase 3/7 positive cells, and found that the % of apoptotic cells was not statistically different between OB-EV-treated and control cells (**Figure 1D**). As expected, osteosarcoma cells that were treated with 5 µM staurosporine (positive control) for the whole duration of the experiment showed a statistically significant increase in apoptosis (**Figure 1D**). Since P53 serves as a critical mediator of several types of apoptosis (35), we assessed its protein expression by western blotting, finding that it was similar in OB-EV-treated and in control cells (**Figure 1E**). Finally, we performed an EdU incorporation assay to evaluate whether proliferative rate was affected by the treatment with OB-EVs, and observed no statistically significant difference when comparing treated and control cells (**Figure 1F**).

### OB-EVs shifted glutathione redox status towards oxidation, without altering lipid peroxidation profile and major ROS-scavenging protection

**Figure 2:**
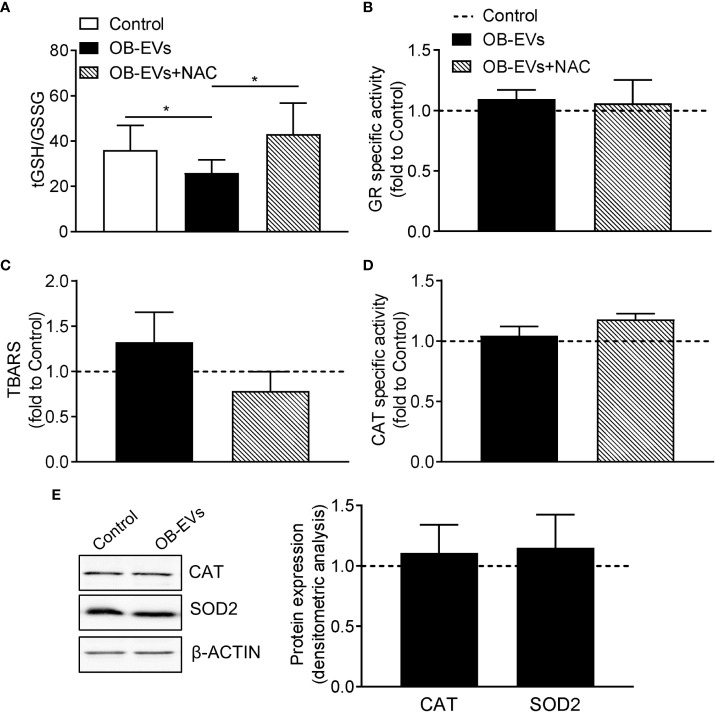
Effects of OB-EVs on redox status, lipid peroxidative damage and antioxidant protection of MNNG/HOS cells. **(A–E)** MNNG/HOS cells were plated and starved in serum-free DMEM O/N the next day. Then, cells were treated with DMEM (control), OB-EVs or OB-EVs + 5 mM N-acetyl-L-cysteine (NAC), for 24 h Cells were subjected to **(A)** quantification of total (tGSH)/oxidised (GSSG) ratio; **(B)** assessment of glutathione reductase (GR) specific activity; **(C)** assessment of TBARS to detect lipid peroxidation; **(D)** evaluation of catalase (CAT) specific activity; **(E)** β-actin-normalised immunoblot-based assessment of CAT and superoxide dismutase 2 (SOD2) protein levels. Representative images of Western blot were reported. **(A)** N=8, **(B, C, E)** N=5, **(D)** N=3. **(A)** Dunn’s test, **(B, C, D)** one-way ANOVA, **(E)** one-sample *t*-test. *p<0.05. Data are presented as mean ± SEM.

As reported above, the antioxidant and cysteine-donor N-acetyl-L-cysteine was able to revert the cell-damaging effects of OB-EVs. Therefore, we measured the redox balance of glutathione, which is an indicator of the antioxidant buffering system within the cell (36), along with the peroxidative damage in all the experimental conditions. Intriguingly, we observed a decreased tGSH/GSSG ratio in OB-EVs-treated MNNG/HOS, as compared to control cells (**Figure 2A**). Interestingly, such a redox perturbing effect was reverted by the co-administration of OB-EVs+NAC (**Figure 2A**). Of note, no OB-EV-dependent effect was observed on the most important enzyme for recycling oxidized glutathione (i.e., glutathione reductase). In fact, the specific activity of GR in OB-EV-treated cells was unchanged, as compared to non-treated cells (**Figure 2B**). Similarly, no change of GR specific activity was detected in cells treated with both OB-EVs and NAC (**Figure 2B**). Surprisingly, the level of lipid peroxidative damage was unchanged by OB-EVs, as shown by the unaltered levels of TBARS (**Figure 2C**). Since catalase (CAT) represents one of the crucial antioxidant enzymes that mitigates cellular oxidative stress, we investigated CAT activity by spectrophotometric enzymatic assay, finding that it was unchanged by OB-EVs (**Figure 2D**), and the same was true for the protein levels of CAT itself and of superoxide dismutase 2 (SOD2), another major mitochondrial antioxidant enzyme (**Figure 2E**). OB-EVs were also analysed with regard to their content in terms of mRNAs relevant to glutathione homeostasis. Our real time RT-PCR analyses revealed that two transcripts that are crucial to glutathione metabolism were undoubtedly present within the OB-EVs. In particular, our presence-absence TaqMan-based assays detected both the glutathione reductase mRNA and the glutamate-cysteine ligase catalytic (*Gclc*) subunit transcript (**Supplementary Figure 1D**).

### OB-EVs did not alter the extension of mitochondrial network, nor did they affect mitochondrial metabolism and dynamics in MNNG/HOS

**Figure 3:**
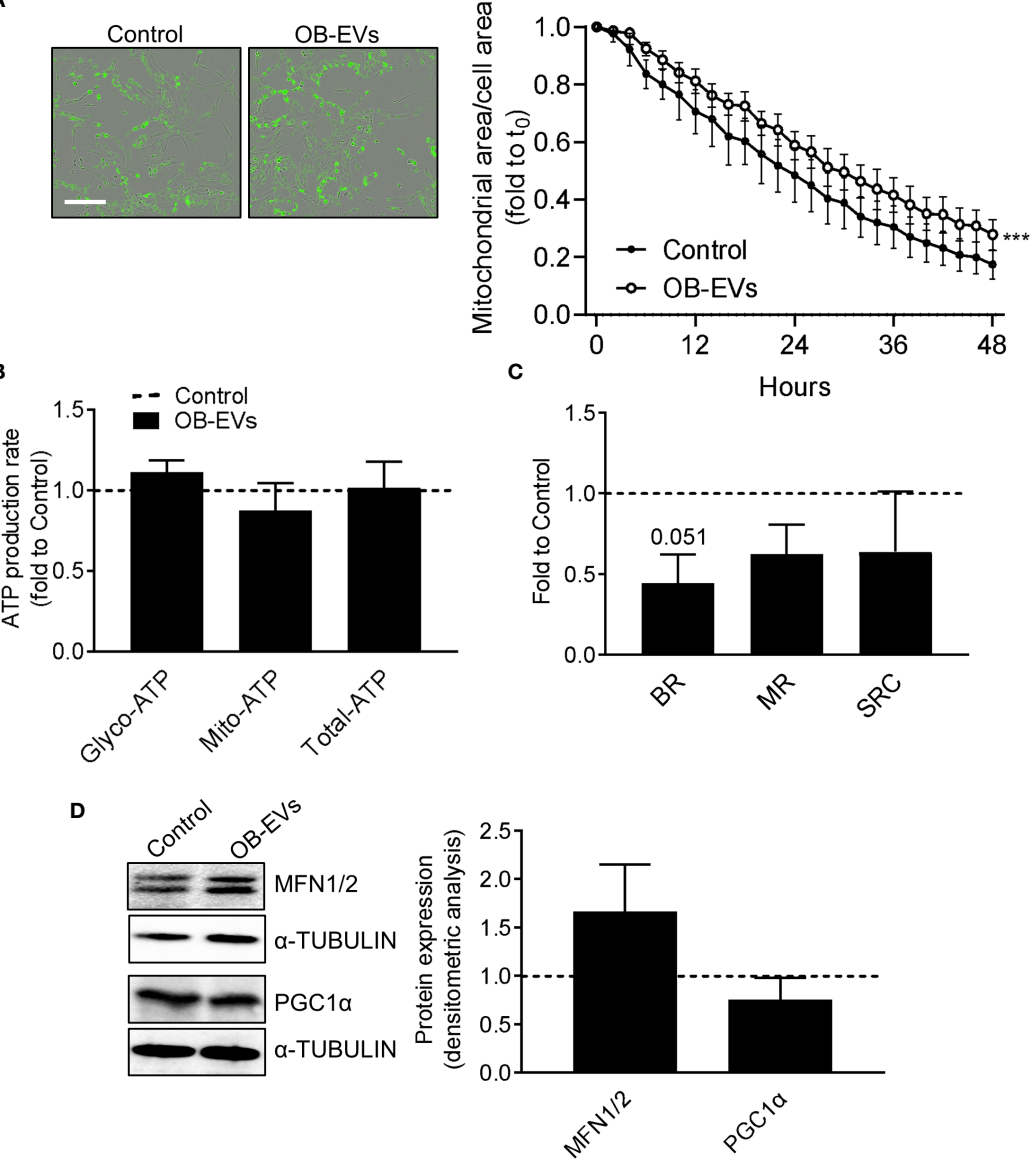
Effects of OB-EVs on mitochondrial function and dynamics of MNNG/HOS cells. MNNG/HOS cells were plated and starved in serum-free DMEM O/N and treated with DMEM (control) or OB-EVs for the time duration specified in the figures, or for 24h when not specified. Then, cells were subjected to **(A)** Mitotracker-based assessment of mitochondrial area/total cell area (scale bar=200 µm); **(B)** SeaHorse-based analysis of glycolytic/mitochondrial/total ATP production rate; **(C)** Mitostress assay to evaluate maximal respiration; **(D)** ɑ-tubulin-normalised immunoblot-based assessment of protein expression of mitofusin 1/2 (MFN1/2) and PGC1α. Representative images of Western blots were reported. **(A)** N=5, **(B–D)** N=3. **(A)** Curve fitting test, **(B, C)** paired *t*-test. ***p<0.001. Data are presented as mean ± SEM.

Since a pro-oxidant perturbation of the redox milieu may be linked to a pro-oxidative switch towards mitochondrial metabolism, we investigated more in detail whether and how mitochondria and cellular energy metabolism could respond to OB-EVs. *Via* a Incucyte-based assay and normalising the Mitotracker-positive area to total cell area, we found that mitochondrial network decreased in OB-EVs-treated osteosarcoma cells less rapidly than in control cells (**Figure 3A**), even though a direct comparison between the mitochondrial area in cells treated with OB-EVs for 24 hours and the corresponding time point in control cells did not reveal any statistically significant difference (**Figure 3A**). Intriguingly, this was not accompanied by a change in the ratio between glycolytic *versus* oxidative ATP produced by MNNG/HOS, as demonstrated by the Seahorse ATP rate assay (**Figure 3B**). However, mitostress assays showed a strong trend of reduction in basal respiration (BR, p=0.051), with non-significant reductions in maximal respiratory capacity (MRC) and spare respiratory capacity (SRC, **Figure 3C**) following OB-EVs treatment. Moreover, the expression level of two important regulators of mitochondrial dynamics (namely, mitofusins 1 and 2) were not significantly affected by the treatment with OB-EVs (**Figure 3D**). Similar results were obtained when we measured the expression level of the master regulator of mitochondrial biogenesis, the peroxisome proliferator co-activator 1 alpha (PGC1ɑ) (**Figure 3D**).

### OB-EVs reduced MNNG/HOS motility and invasion

**Figure 4:**
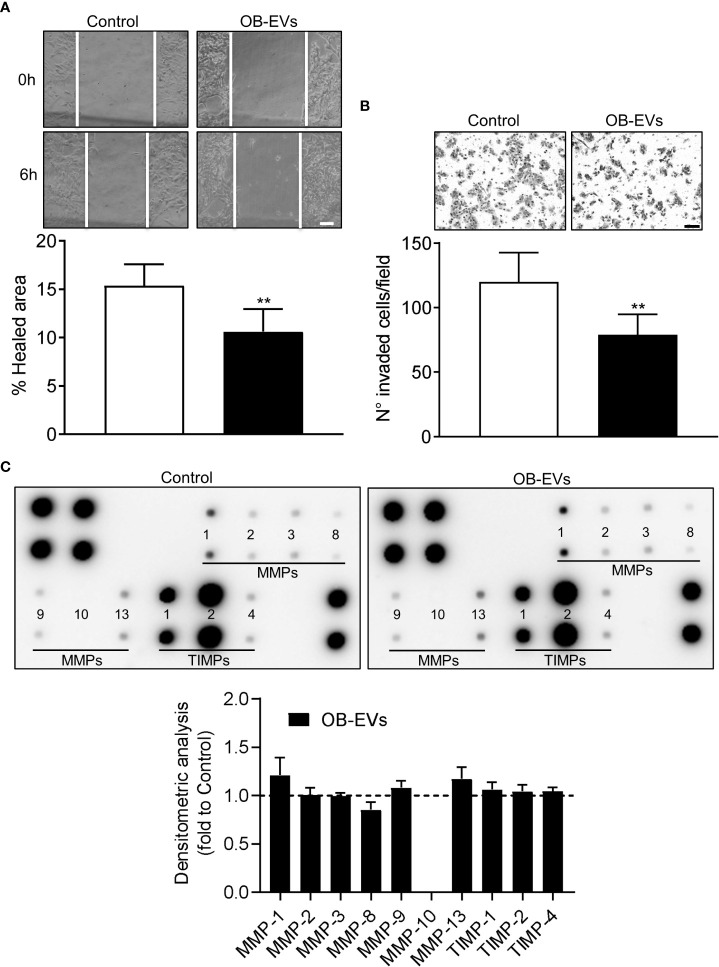
Effects of OB-EVs on motility and invasiveness of MNNG/HOS cells. MNNG/HOS cells were plated and starved in serum-free DMEM O/N the next day. Then, cells were treated with DMEM (control) or OB-EVs for 24 hours, **(A)** plated in 24 well plates to perform a scratch assay (scale bar=100 μm) or **(B)** seeded on the upper basket of a matrigel-coated transwell to perform an invasion assay over FBS (scale bar=100 μm). **(C)** Conditioned media (CM) were collected from DMEM- or OB-EVs-treated MNNG/HOS after 24 hours of treatment and subjected to metalloproteinase (MMPs) antibody array. Representative images of membranes incubated with CM from control or OB-EV-treated MNNG/HOS cells, and evaluation of secreted proteins (MMP-1, -2, -3, -8, -9, -10, -13, TIMP-1, -2, -4), as determined by densitometric analysis of spots of interest that were normalised for the positive control spots and shown as fold to control. **(A)** N=6, **(B)** N=5, **(C)** N=3. Paired Student’s *t*-test. **p<0.01. Data are presented as mean ± SEM.

A key feature of aggressive cancer cells is the ability to migrate linearly and invade basal membranes. To check if OB-EVs affected these characteristics, we pre-treated MNNG/HOS with EVs for 24 hours, then we assessed scratch wound healing and invasion assays to evaluate *in vitro* tumour cells motility and invasiveness, respectively. Interestingly, both functions were reduced by OB-EVs treatment (**Figures 4A, B**). However, metalloproteinases (MMPs) arrays ran on conditioned media of MNNG/HOS cells treated with OB-EVs showed no difference *versus* untreated MNNG/HOS (**Figure 4C**).

### OB-EVs did not affect MNNG/HOS primary oncospheres formation

**Figure 5:**
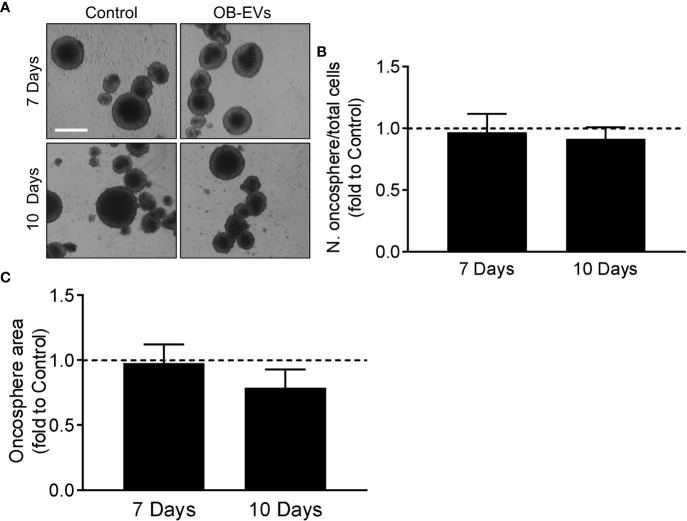
Effects of OB-EVs on stemness phenotype of MNNG/HOS cells. **(A–C)** MNNG/HOS cells were detached, reduced into a single-cell suspension and plated at a density of 2,000 cells/ml in oncosphere medium on poly-hydroxyethyl-methacrylate-coated plates and in a specifically-formulated medium to favour the formation of oncospheres, marker of stemness phenotype. Cells were then treated with DMEM (control) or OB-EVs twice a week. After 7 and 10 days, **(A)** pictures of the oncospheres were taken (scale bar=200 μm) to analyse their **(B)** number/plated cells ratio and their **(C)** total area. One sample *t*-test, N=4. Data are presented as mean ± SEM.

Another important characteristic of aggressive cancer cells is their *in vitro* stemness, which is an indication of how many tumour-initiating cells are present in the population of cancer cells. We assessed this by primary oncosphere formation assay (**Figure 5A**), but we found no differences in their number/seeded cells (**Figure 5B**) or total area (**Figure 5C**) of the spheroids in cells treated with OB-EVs *vs* control cells.

## Discussion

Osteosarcoma is still a largely understudied neoplasia. The chances of survival for patients dramatically drop when metastases, preferentially to the lungs, develop (3) and therapy resistance occurs (6). Such disease progression is often achieved through the cross-regulation between osteosarcoma cells and bone cells in the TME, and EVs are emerging as key players in this regulatory interplay. Indeed, in a previous work, we demonstrated that MNNG/HOS-derived EVs influence osteoblasts, by reducing their differentiation and increasing their release of pro-inflammatory cytokines (20). Here, we wondered if also osteoblasts could signal back to osteosarcoma cells through EVs. Therefore, as a first basic approach, we aimed at evaluating whether OB-EVs could affect proliferation dynamics and viability in MNNG/HOS cells. As shown by the EdU-based approach, the proliferation rate of MNNG/HOS cells was unchanged upon treatment with OB-EVs. Conversely, we found that OB-EVs reduced the viability of MNNG/HOS cells, and this was observed using two different methods (i.e. Trypan blue-exclusion test and MTT assay). In order to verify whether such a reduction in cell viability was caused by increased apoptotic death, we measured caspase 3/7-dependent cell death by time-course fluorescence microscopy, and found no evidence of any change in casp3/7-dependent apoptotic rate in osteosarcoma cells treated with OB-EVs. Consistently, P53 protein levels were not affected by the treatment with EVs. This would suggest that the exposure to OB-EVs could induce necrotic death or even non-classical apoptosis in MNNG/HOS cells. In fact, despite the fact that it is generally acknowledged that members of the caspase family of proteases play a pivotal role in the execution of apoptotic cell death (37), the existence of caspase-independent forms of programmed cell death (PCD) has been proved in the recent past (38–40). This intriguing result deserves further investigation to clarify the exact pathway activated by the exposure of osteosarcoma cells to OB-EVs, with particular focus on the redox-responsive non-classical forms of PCD, such as necroptosis, which has been recently proposed as an inducible pathway to activate an ordered form of cell death in osteosarcoma cells and osteosarcoma stem cells (41, 42).

The next step was to investigate two key features that often predict aggressiveness and tumorigenicity of cancer cells, such as migration and invasion capacities. With regard to this aspect, we describe here that OB-EVs reduced cellular motility and invasion capacity, even though no changes in the expression of the major matrix-degrading enzymes were detected. Taken together, these observations corroborate the idea that OB-EVs diminished the ability of osteosarcoma cells to invade the surrounding tissues, but this was probably not achieved by decreasing cellular capacity to digest matrix and basal membranes, but rather by reducing their overall ability to move. However, we cannot rule out the possibility that OB-EVs impaired some form of MMP-independent invasion process. Indeed, some authors have described that inhibitors of the proteolytic activity of MMPs were not able to prevent invasion of immortalised mouse epithelial cells through a sparse three-dimensional collagen matrix (43). Further experiments will attempt to clarify this aspect more in detail.

It is well-established that a subgroup of cancer cells, including osteosarcoma, is able to self-renew and initiate tumours *in vivo*. These are commonly termed cancer stem cells (CSCs), or tumour-initiating cells (TICs) (44, 45). CSCs have also been reported to present lower levels of ROS and a different redox balance compared to their non-stem counterparts (46), and we therefore deemed it interesting to evaluate whether OB-EVs would change the stem-like phenotype of osteosarcoma cells. To this end, we used the oncosphere formation assay, since the ability to form spheroids is directly associated with the stem cell-like phenotype in osteosarcoma (44, 47). However, our analysis showed that no differences were caused by treatment with OB-EVs, and control cells were able to form a similar number of similarly-sized spheroids.

The results presented so far suggested that OB-EVs elicited a clear cytotoxic effect in MMNG/HOS cells, significantly reducing the major traits of their malignant behaviour. In this context, redox homeostasis is increasingly considered as an essential resilience trait that helps cancer cells to preserve viability and growth capacity, along with resistance to external stressors (48–50). In particular, reactive oxygen species (ROS) serve as signalling entities in cells, regulating key cellular processes such as proliferation, survival, and adaptive response to external stimuli, as broadly reviewed by Sies and Jones (51). This is not only true in physiological conditions, but also in hyperproliferation-based diseases, such as in tumours and cancers (52). On this basis, we wanted to investigate whether and how OB-EVs could cause biochemical and molecular alterations in the balance of the redox milieu of osteosarcoma cells. Here we show that a 24-hour treatment with OB-EVs significantly decreased the total/oxidised glutathione. Glutathione is a small endogenous thiol-containing tripeptide that in its reduced form (GSH) fuels direct and indirect ROS-scavenging antioxidant reactions within cells (53). Upon being used as a reducing molecule, GSH is promptly converted to glutathione disulfide (GSSG), therefore a decrease in the total/oxidised glutathione in a biological system indicates either that an over-production of pro-oxidants is occurring or that an impaired biosynthesis/recycling of glutathione is taking place (54, 55). In order to exclude that the recycling pathway was involved in such OB-EV-induced impairment, the specific activity of glutathione reductase (GR), the most important enzyme for recycling GSSG within cells (56) was assayed, and we found evidence that the enzymatic function of GR in OB-EV-treated cells was unaltered, nor was it changed by treatment with both OB-EVs and NAC, thus pointing out to the *de novo* formation of GSH as the redox-active biosynthetic pathway possibly involved in the pro-oxidant effect of OB-EVs on human osteosarcoma cells. This working hypothesis will be verified in the next future, by more specific experiments focused on evaluating the expression of the rate-limiting enzyme responsible for GSH synthesis (i.e., glutamate cysteine ligase). However, it should be also noted that cancer cells divert glucose utilisation to the pentose phosphate pathway (PPP) to support cell survival and growth by generating building blocks for nucleic acid synthesis, along with the NADPH needed for fatty acid synthesis and cell survival under stress conditions (57). Therefore, the possibility that the treatment with OB-EVs could alter PPP activity, thus down-regulating the synthesis of NADPH in osteosarcoma cells, will certainly be considered in our next work. Importantly, we also demonstrated that EVs isolated from osteoblasts included both the glutathione reductase and the *Gclc* subunit transcripts, thus further confirming that the glutathione redox homeostasis of the cells interacting with the vesicles might be altered.

More in general, the shift of the glutathione redox equilibrium towards a more oxidised state might indicate the occurrence of an oxidative stress condition (54, 55). On this basis, we hypothesised that OB-EVs may elicit a pro-oxidant effect on osteosarcoma cells. The fact that OB-EVs served as a ROS-dependent stimulus on MMNG/HOS cells was confirmed by the observation that the co-incubation with the ROS-scavenging compound and cysteine-donor N-acetyl-L-cysteine (NAC) was able to revert completely the pro-oxidant effect of OB-EVs in terms of glutathione redox balance. Interestingly, NAC also inhibited the cytotoxic effect of OB-EVs, thus strongly suggesting that the cell death induced by OB-EVs was dependent on the ROS-promoting effect of extracellular vesicles.

In order to further investigate more in detail how osteosarcoma cells were challenged by OB-EVs *via* ROS-dependent mechanisms, we measured the specific activity of catalase, which is known to play a critical role in first line defence against oxidative stress within cells (55, 58, 59). Our experiments revealed that the treatment with OB-EVs did not affect the specific activity of catalase, nor did the treatment change CAT protein expression level. Similar results were obtained when the protein level of another important antioxidant enzyme (namely, the superoxide dismutase 2) was studied. SOD2 and CAT are inducible enzymes whose up-regulation upon oxidative stress represents a critical step in the adaptive response of cells to pro-oxidant stimuli, at mitochondrial and peroxisomal level, respectively (47, 60–63). Taking into account the pivotal role played by mitochondria and peroxisomes in the regulation of the redox metabolism in the eukaryotic cell (64), the findings we present here suggest either that the stress level induced by OB-EVs may have cytotoxic effect before any adaptive response in osteosarcoma cells could initiate, or that the OB-EV-dependent cytotoxicity could rely more on a mild imbalance of redox homeostasis rather than on severe oxidative stress condition. In order to settle this, we measured the levels of TBARS, which is a well-established marker of lipid peroxidative molecular damage (65). Our experiments did not reveal any OB-EV-induced change in the TBARS levels. This suggests that the treatment with OB-EVs could activate a redox-dependent cell death pathway in osteosarcoma cells without initiating a state of full-blown ROS-based molecular damage. This seems to be confirmed by the evidence that mammalian catalase, which intervenes in the antioxidant defence when the level of oxidative stress within cells is severe (66), was unaffected by the treatment with OB-EVs. On this basis, our results suggest that OB-EVs may act as a cytotoxic stressor for osteosarcoma cells more through a modest imbalance of redox homeostasis rather than *via* promoting an oxidative stress condition. However, it cannot be ruled out that assessments at different time points might reveal a pattern of tentative antioxidant response, at least at the enzymatic level. Nor can we rule out the possibility that OB-EVs could cause oxidative-dependent damage to macromolecules other than lipids. Such hypotheses will be clarified by more targeted experimental approaches in the future.

Mitochondrial redox-active metabolism and ROS-promoting enzymatic reactions, along with ATP generation, seems to serve as a crucial determinant for malignant phenotype and anticancer drug-resistance (48, 67, 68). Moreover, even though many cancers exhibit increased rates of glycolytic catabolism, increasing proofs support the idea that mitochondria remain a crucial source of ATP, and serve as key organelles that integrate a plethora of metabolic and signalling pathways in tumours and malignancies (69). On this basis, we wanted to verify whether OB-EVs could also alter the function and the extension of the mitochondrial network in osteosarcoma cells. Our Mitotracker-based investigation revealed that cell growth determined a decline in the extension of the mitochondrial compartment in both OB-EVs-treated and control osteosarcoma cells, and this was most likely due to the growth of the cytoplasm that accompanied cell growth. However, in OB-EVs-treated cells the decline of the mitochondrial network over the timeframe of 48 hours was less rapid. This could be due to the inhibiting effect of OB-EVs on cell growth, or a compensatory mechanism that counteracts a possible detrimental effect on mitochondria. Most importantly, it should be noted that the difference between OB-EVs-treated and control at 24h, *i.e.* the time at which all other assays were performed, was not statistically significant. Although OB-EVs did not change the glycolytic/OxPhos ATP ratio, MitoStress experiments revealed an almost significant trend (p=0.051) of reduction in basal respiration, and a reduction in maximal respiration and spare respiratory capacity in 3 over 4 experiments analysed in OB-EVs-treated cells. Considering the significance of such parameters (70), this result might suggest that OB-EV-treated osteosarcoma cells could have a lower threshold below which the basal ATP demands cannot be satisfied. This may be important, as the exposure to OB-EVs could weaken osteosarcoma cells towards further exogenous stressors, with clear repercussions on oncology research and clinical treatments.

Reprogramming of mitochondrial activity is thought to underlie chemoresistance and metastatic behaviour (69). In addition, mitochondrial fusion and biogenesis, which participate to the so-called mitochondrial dynamics, dictate mitochondrial morphology and function (71–73), influence the redox homeostasis and antioxidant defence of cancer cells, along with their apoptotic response to OS-promoting chemotherapeutics (74). On this basis, we investigated whether the expression of the major controllers of mitochondrial fusion and biogenesis was affected by OB-EVs, however no evidence was found for such an effect, as shown by the unaltered protein levels of MFN1/2 and PGC1ɑ. MFN1/2 and PGC1ɑ are key players belonging to the fine-tuned molecular pathway that govern mitochondrial dynamics (75). In this context, our results clearly suggested that the redox-dependent cytotoxic effect elicited by OB-EVs on osteosarcoma cells was not associated with any major re-organisation of the mitochondrial compartment. This finding was substantially confirmed by the IncuCyte-based analysis of the extension of the mitochondrial network. In fact, we observed that a 24-h treatment with OB-EVs did not significantly change the area of the mitochondrial network. As expected, our investigation revealed by fluorescence microscopy that the Mitotracker-positive area decreased in a time-dependent fashion. This was most likely due to cell growth over the time. However, our observations revealed that in OB-EV-treated osteosarcoma cells the Mitotracker-positive area decreased over time less rapidly than in control cells. This could be due to the cytotoxic action of OB-EVs that inhibited the growth of the confluence phase area in treated cells.

In conclusion, our findings suggest that osteoblast-derived extracellular vesicles could be an important means of cross-communication between the TME and the osteosarcoma core. For the first time to the best of our knowledge, we proved that OB-EVs reduced osteosarcoma cells’ aggressiveness and viability through redox-dependent signalling pathways. However, we did not find strong evidence of the involvement of changes in mitochondrial dynamics or major energy metabolic switch in the phenotypic change induced by osteoblast-derived extracellular vesicles in osteosarcoma cells. It should be noted that OB-EVs were isolated from osteoblasts that are not being co-cultured with osteosarcoma cells, therefore representing a model of “healthy” osteoblasts, whereas we demonstrated (20) that MNNG/HOS-EVs cause devastating effects on them, thus changing their phenotype. Hence, we could speculate that keeping osteoblasts ``healthier” by preventing the effect of HOS-EVs on them, would result in reduced osteosarcoma aggressiveness. Although this has not been observed in osteoblasts, similar effects are exerted by mesenchymal stem cells-derived EVs (MSC-EVs), which are closely related to osteoblast and can differentiate into them (76). In fact, MSC-EVs reduce osteosarcoma aggressiveness when harvested from healthy MSCs (77, 78), and conversely, they promote osteosarcoma aggressiveness when co-cultures between the two cell types are established (78, 79).

Should the pathophysiological relevance of this phenomenon be confirmed *in vivo*, osteoblasts could be exploited as endogenous anti-cancer weapons, as proposed for leukaemias by other researchers (80).

## Data availability statement

The raw data supporting the conclusions of this article will be made available by the authors, without undue reservation.

## Ethics statement

The animal study was reviewed and approved by Ministero della Salute italiano (Italian Ministry of Health).

## Author contributions

MP: Methodology, investigation, data curation, visualisation, software, writing - original draft, writing - review & editing. AU: Methodology, investigation, data curation, visualisation, writing-review & editing. CP: Methodology, investigation, data curation. LG: Methodology, investigation, data curation. IF: Methodology, investigation, data curation. DC: Conceptualisation, methodology, investigation, data curation, resources. FZ: Conceptualisation, data curation, writing - review & editing. AC: Conceptualisation, data curation, methodology, review & editing. NR: Conceptualization, supervision, funding acquisition, methodology, investigation, writing - original draft, writing - review & editing. SF: Conceptualization, supervision, funding acquisition, methodology, investigation, writing - original draft, writing - review & editing. All authors contributed to the article and approved the submitted version.

## Funding

This work was supported by: i) The Associazione italiana per la ricerca sul cancro (AIRC) Investigator Grant #24823 to NR; ii) The AIRC fellowship for Italy #25432 to MP for salary; iii) the competitive grant for Basic Research of the University of L’Aquila (D.R. no. 649/2022).

## Acknowledgments

We wish to thank Dr. Lorenzo Arrizza for his priceless help and assistance in TEM analysis.

## Conflict of interest

The authors declare that the research was conducted in the absence of any commercial or financial relationships that could be construed as a potential conflict of interest.

## Publisher’s note

All claims expressed in this article are solely those of the authors and do not necessarily represent those of their affiliated organizations, or those of the publisher, the editors and the reviewers. Any product that may be evaluated in this article, or claim that may be made by its manufacturer, is not guaranteed or endorsed by the publisher.
