# Supplementary material for: Effects of osteoblast-derived extracellular vesicles on aggressiveness, redox status and mitochondrial bioenergetics of MNNG/HOS osteosarcoma cells
Source: Front Oncol. 2022 Dec 5;12:983254. doi: 10.3389/fonc.2022.983254 (PMC9762506; doi:10.3389/fonc.2022.983254)
Supplement: Supplementary file 1 [file DataSheet_1.pdf]

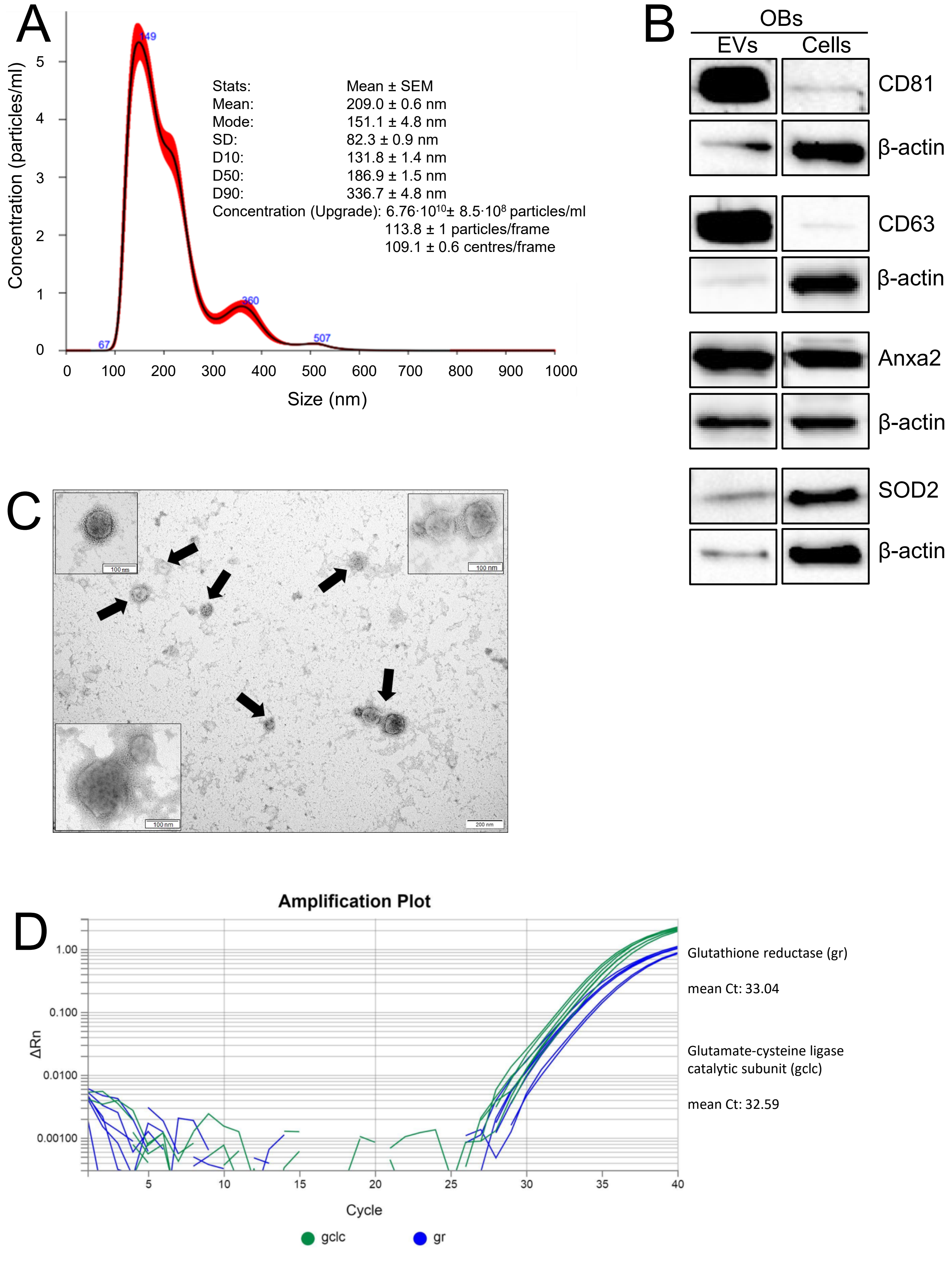

Supplementary Fig.1

SUPPLEMENTAL MATERIAL

Supplementary Figure 1. Characterisation of osteoblast-derived EVs. Mouse primary osteoblasts were starved in serum-free DMEM for 24 hours and EVs were isolated from their conditioned medium (CM) by ultracentrifugation. **(A)** Size and concentration determination of OB-EVs (isolated from 12 ml of CM collected from one 175 cm^2 flask, cel l density = $3.5x10^4$ cells/cm^2) by nanoparticle tracking analysis (NanoSight NS300). **(B)** Western blot for the EV positive markers CD81, CD63, Annexin II (Anxa2), for the negative marker SOD2 and for β-Actin as loading control in protein lysates (8µg) extracted from osteoblasts (OBs) and osteoblast-derived EVs (OB-EVs). **(C)** Transmission Electron Microscopy (TEM) evaluation of OB-EVs (arrows), original magnification 34000x; insets: higher magnification (92000x) in different fields of the same EVs sample. Data are representative of 3 independent preparations. **(D)** TaqMan-based real time RT-PCR amplification plot of glutathione reductase (gr) and glutamate-cysteine ligase catalytic subunit (gclc) transcripts in total RNA isolated from OB-EVs. Data are from 2 independent preparations.

Supplementary Table I: Antibodies and dilutions used for Western blot analyses.

Target	Manufacturer	Catalogue #	RRID#	Working dilution
β-actin	Santa Cruz Biotechnologies	sc-81178	AB_2223230	1:1000
Annexin II	Santa Cruz Biotechnologies	sc-9061	AB_2057340	1:200

CD81	Santa Cruz Biotechnologies	sc-166029	AB_2275892	1:400
CD63	Thermo Fisher Scientific	PA5-92370	AB_2806456	1:500
CAT	Abcam	ab16731	AB_302482	1:2000
SOD2	Abcam	ab86087	AB_2191662	1:1000
p53	Cell signaling technologies	48818S	AB_2713958	1:1000
MFN1/2	Abcam	ab57602	AB_2142624	1:750
PGC1ɑ	Santa Cruz Biotechnologies	sc-518025	AB_2890187	1:200
ɑ-tubulin	Sigma Aldrich	T5168	AB_477579	1:4000
**Secondary antibodies (HRP conjugated)**				
Goat anti-mouse IgG	Thermo Fisher Scientific	31430	AB_228307	1:5000
Mouse anti-rabbit IgG	Santa Cruz Biotechnologies	sc-2357	AB_628497	1:5000

Abbreviations: CAT, catalase; MFN1/2, mitofusin 1/2; PGC1ɑ, Peroxisome proliferator-activated receptor gamma coactivator 1-alpha; SOD2, superoxide dismutase 2.
